# Investigation into the Value of Trained Glycaemia Alert Dogs to Clients with Type I Diabetes

**DOI:** 10.1371/journal.pone.0069921

**Published:** 2013-08-07

**Authors:** Nicola J. Rooney, Steve Morant, Claire Guest

**Affiliations:** 1 Anthrozoology Institute, University of Bristol, Bristol, United Kingdom; 2 Medicines Monitoring Unit, University of Dundee, Dundee, United Kingdom; 3 Medical Detection Dogs, Greenway Business Park, Milton Keynes, United Kingdom; University of Sussex, United Kingdom

## Abstract

Previous studies have suggested that some pet dogs respond to their owners’ hypoglycaemic state. Here, we show that *trained* glycaemia alert dogs placed with clients living with diabetes afford significant improvements to owner well-being. We investigated whether trained dogs reliably respond to their owners’ hypoglycaemic state, and whether owners experience facilitated tightened glycaemic control, and wider psychosocial benefits. Since obtaining their dog, all seventeen clients studied reported positive effects including reduced paramedic call outs, decreased unconscious episodes and improved independence. Owner-recorded data showed that dogs alerted their owners, with significant, though variable, accuracy at times of low and high blood sugar. Eight out of the ten dogs (for which owners provided adequate records) responded consistently more often when their owner’s blood sugars were reported to be outside, than within, target range. Comparison of nine clients’ routine records showed significant overall change after obtaining their dogs, with seven clients recording a significantly higher proportion of routine tests within target range after obtaining a dog. HbA1C showed a small, non significant reduction after dog allocation. Based on owner-reported data we have shown, for the first time, that trained detection dogs perform above chance level. This study points to the potential value of alert dogs, for increasing glycaemic control, client independence and consequent quality of life and even reducing the costs of long-term health care.

## Introduction

Diabetes is a chronic condition affecting 2.6 million people in the UK, approximately 10% of which have Type I diabetes [1]. Hypoglycaemia is a common and potentially life threatening complication of diabetes in individuals receiving insulin and is especially hazardous for long-term patients who may have lost the ability to recognise early warning signs. Unawareness has been reported in 25% of Type 1 diabetes patients, increasing their risk of severe hypoglycaemic episodes by 6–7 fold [2].

Fear of hypoglycaemia amongst patients with diabetes has been reported to be as great as that of blindness and renal failure [3]. Hence individuals often restrict their lifestyle to reduce the risk of hypoglycaemic events: thereby profoundly reducing their psychological wellbeing and quality of life [4]. Some also manipulate injected insulin levels which can increase the risk of long-term deleterious consequences of hyperglycaemia [2]. Thus, there is great potential value in an early warning system which alerts patients to impending lowered blood sugars. Yet despite considerable resources having been invested in developing electronic systems to facilitate tightened glycaemic control, current equipment has numerous limitations (e.g. [5]).

Recently, the dog has been suggested as a “biocompatible and patient friendly alarm system” for hypoglycaemia [6]. Case studies have indicated that pet dogs can spontaneously exhibit specific behaviours when their owner’s blood sugars decrease, most commonly vocalising, nuzzling, licking, biting, jumping up and staring at their owner [7], [8]. Reports describe individual dogs alerting their owners during nocturnal episodes (which can be especially dangerous and are particularly feared [5]): repeatedly reacting to hypoglycaemia whilst their owner is driving [9] and consistently responding to hypoglycaemia in a non-diabetic owner [10]. When interviewed, 38% of dog-owning patients with diabetes (N = 37) reported changes in their pet dogs’ behaviour during hypoglycaemia [11]. Two larger written surveys reported 68% (N = 304; [7]) and 65% (N = 212; [8]) of dogs similarly responding. Whilst these studies indicate that the capacity for dogs to respond to hypoglycaemia is extremely likely, written and verbal survey results differ, and all studies relied on owners accurately recalling past events. Hence the frequency with which dogs respond may be over-reported.

Following anecdotal evidence of spontaneous responses, charities have started to train dogs to systematically alert owners with diabetes. The first organisation to train extensively in the UK is Medical Detection Dogs (MDD; http://medicaldetectiondogs.org.uk) which to date has trained over twenty dogs. Some dogs are already owned by clients and their behaviour is shaped by professional trainers, others are specifically procured, trained, and placed with pre-selected clients. Using principles of associative learning, dogs are rewarded for showing “alerting” behaviours when their owner’s blood sugar levels fall outside a specifically agreed target range, usually 5–15 nm/l, but varying slightly dependent upon the specific client’s needs. Trained alerting behaviours include licking, pawing, jumping, staring, vocalising and even fetching a blood testing kit. Charities claim that the dogs are effective at facilitating tightened glycaemic control, and thus reducing hypoglycaemic episodes, nocturnal lows and paramedic call outs. This study is the first to assess these claims empirically.

Via client interviews, we aimed to collect information on the perceived value of trained dogs and their effects on glycaemic control. We assess whether dogs reliably alert their owners, by comparing glucose levels during routine sampling (approximately six times daily) to those whenever the dog has “alerted”. We address the questions, “Is there evidence that alert samples were more likely to be out of range than routine samples?”, and, “Does this vary between dogs?”. We then examine whether glycaemic control is improved once people have a trained dog, by comparing routine blood records for before and after dog allocation (or training if an existing companion dog was trained), hypothesising that the proportion of samples within the target range will increase. We also look specifically at the incidence of nocturnal low sugars, pre and post-dog.

Whilst previous studies of dogs have concentrated on hypoglycaemic episodes exclusively, we also examine hyperglycaemia. Some dogs start to respond spontaneously at times of high blood sugar and, hence, many dogs have now been trained to alert at such times. This is important, as there is limited value to an intervention which decreases the number of hypoglycaemic episodes, yet increases the client’s time in hyperglycaemia. HbA1C (glycated haemoglobin) correlates to the average plasma glucose concentration over prolonged periods giving an indication of long-term tightness of glycaemic control. Reducing HbA1C, whilst also minimising the time in hypoglycaemia, is therefore a goal in the management of diabetes [12], [13], so we compare HbA1C medical records from before and after dog allocation.

## Methods

### Ethical Statement

Ethical approval was granted by the University of Bristol, Faculty of Medical and Veterinary Sciences Ethics Committee, and written informed consent obtained from subjects and, from next of kin on the behalf of the minors/children participants, for each stage of this study.

### The Subjects

Nineteen clients of Medical Detection Dogs, with fully trained and certified (N = 11), or advanced trainee dogs (N = 8; deemed by their trainer to be performing their alerting function well, although not yet having done so for a full three months, or achieved adequate general obedience as required for certification) were contacted by letter to request their participation in the study. Two clients declined due to current hospitalization or recent bereavement. The remaining seventeen comprised four males and 13 females (Table 1): aged between five and 66 years (median 41 years). Three were children so interviews were conducted with a parent; 3a and 3b were father and son utilising the same alert dog so only the father was interviewed. Client 4 had Type II diabetes whilst the remainder had Type I. Clients had lived with a trained dog for between four months and seven years (average± standard deviation = 1.9±1.9 years).The dogs comprised six Labrador Retrievers (LR), one Golden Retriever (GR), two LR/GR cross, one Poodle, one Collie Cross, two Labradoodles, one Lurcher, one Cocker Spaniel and one Yorkshire Terrier.

**Table 1:** Details of the clients and data each provided.

Client number	Age (years)	Dog owned by clientand trained in situ (O) or trained and placedby MDD (T)	Time since acquisition(in years)	Dog qualified attime of study	Routine/alert comparison	Pre/postdog routinecomparison	HB1c data	Reported alerting behaviours
1	52	O	0.75	0	1	1	1	stare, paw & sometimes grunts
2	35	O	1.17	1	1	0	1	yawn, tap me & stay with me
3a*	41	T	1.1	1	1	0	1	pushing hand & lick, focused & intense, making a fuss jumping up
3b*	8	T	as above	1	1	1	0	as above
4	59	O	2.5	0	0	0	1	looking, licking & climbing up
5	55	O	0.67	1	1	1	1	agitated then stares
6	44	O	7	1	0	0	0	squeak & sit next to me, if other person present alerts them though not trained to
7	7	T	1.42	1	1	1	1	licking & not leaving me
8	17	T	0.75	0	1	1	0	licks hard or stares, squeak, whines & gets blood checking kit on instruction
9	49	O	1.33	0	0	0	1	starts licking & sniffing, anxious, circles, goes to treat drawer and comes back, if don’t respond stares & barks
10	66	O	4	1	1	1	1	jumps up, licks sits next to
11	43	T	5.40	1	0	0	1	jumps up & barks, hits emergency button, if pass out or if I tell him “touch”
12	5	T	1	0	0	0	0	goes to mum & puts head on her, if she ignores nudge & climb up & paws. If asleep licks 7 climbs on mum
13	50	O	1.5	1	1	1	1	bring test kit, then sniffs mouths & face, if ignore, climbs up, licks & barks
14	31	T	0.67	0	0	0	1	licks hands & now trained to jump up
15	41	T	0.34	0	1	1	0	jumps up
16	29	T	0.75	0	1	1	0	sits, licks, sniffs, paws my leg. At night lies on the bed & brings testing kit

* two family members both using same alert dog.

### The Visit

Each client was visited in their own home by the main researcher (NJR). Visits took approximately ninety minutes and included a structured interview with thirty four questions collecting information on clients’ experiences with diabetes, opinions of the value of their dog, and the frequency with which they recalled hypoglycaemia-related events prior to, and after acquiring the dog. For clients whose own dog was trained, we refer to dog acquisition/allocation as the time when the dog began alerting reliably. Many responses are reported separately [14], so only those relevant to the current aims are reported here. We verbally presented each client with ten statements (Table 2) regarding the impact of the dog upon their life and they were asked to rate (on a five point scale) the extent to which they agreed with each.

**Table 2:** Extent to which each client reported agreeing with each of ten statements regarding the effect of the dog upon their lives.

	5	4	3	2	2
Number of clients reporting each category	totally agree	somewhat agree	neither agreenor disagree	somewhat disagree	totally disagree
I am more independent since I obtained my dog	12	2	2	0	0
Having a trained dog is a big commitment	5	6	1	2	2
The dog has enhanced my quality of life	13	2	1	0	0
There are disadvantages of having an alert dog	0	0	4	4	8
I enjoy the conversations which the dog’s coat promotes	5	4	7	0	0
I trust my dog to alert me when my sugarlevels are low	11	3	1	0	1
I dislike the attention which the coat attracts	0	2	4	2	8
I am totally satisfied with my dog	13	2	1	0	0
If I had my time again, I wouldn’t get a dog	0	0	0	1	15
I trust my dog to alert me when my sugarsare high	6	7	0	1	2

Underlined cells denote the most popular responses for each statement.

Each client was requested to take part in the next phase of the study: recording their dog’s alerting behaviour and providing routine blood test results. All but one agreed, and also granted written permission to access their routine blood test results, given to the charity pre-dog allocation (approximately one month’s worth recorded shortly before dog acquisition), and to contact their diabetes clinic and/or general practitioner to obtain HbA1c records, from routine 6-monthly diabetes checks. All HbA1c records post-dog allocation were requested (1–9 per client), along with at least two records pre-dog; clinics provided between two and 23 records per client. Clients were given data collection forms on which to record the date, time and location every time they took a blood glucose reading. They were urged to continue their usual testing regime, and to record all tests and alerts. They also recorded whether each reading was routine, or made in response to the dogs’ alerting behaviour. If an alert, they described the dog’s behaviour and their own activity at the time. Clients were requested to keep records for at least a month, although the number of records kept varied greatly (Table 3). Clients were informed of the study’s latter aim: to analyse how dogs performed in different situations, in order to optimise future training. This knowledge is likely to have decreased their temptation to exaggerate accounts of their dog’s success.

**Table 3:** Distribution of glucose concentrations with respect to each client’s target range in routine blood samples taken before and after the acquisition of their dog.

Client	Target range (nm/l)	Acquisition	Samples	Distribution of samples (%)				p-values (comparing before and after acquisition)			Mean number of blood samples per day					
				Very low (<2.5 nm/l)	Low	Within range	High	Overall Distribution^1^	% very low^2^	% within range^2^	Routine	p-value3 compare before and after acquisition	Routine and alert		p-value^3^	
1	4.5–10	Before	577	3.8	13.7	52.0	30.5				7.0					
		After	248	1.2	8.1	49.6	41.1	0.002	0.047	0.514	5.2	<0.001		6.9		0.886
3B	5–15	Before	312	3.8	32.7	56.4	7.1				4.5					
		After	695	2.9	18.4	63.2	15.5	<0.001	0.439	0.043	5.3	0.017		6.6		<0.001
5	5–15	Before	271	3.3	43.5	51.3	1.8				3.0					
		After	76	.	40.8	56.6	2.6	0.372	0.215	0.438	2.4	0.004		2.2		<0.001
7	5–15	Before	386	1.8	11.9	44.3	42.0				5.0					
		After	255	3.5	14.5	63.1	18.8	<0.001	0.200	<0.001	3.7	<0.001		4.0		<0.001
8	5–10	Before	128	3.1	29.7	48.4	18.8				4.6					
		After	422	0.2	10.9	61.8	27.0	<0.001	0.012	0.008	3.4	<0.001		4.2		0.026
10	5–15	Before	416	5.0	24.3	65.9	4.8				6.2					
		After	283	2.1	8.5	76.7	12.7	<0.001	0.070	0.002	3.5	<0.001		7.0		0.016
13	5–15	Before	240	1.7	17.9	37.1	43.3				2.9					
		After	227	.	0.4	99.1	0.4	<0.001	0.124	<0.001	2.5	0.078		5.4		<0.001
15	4–15	Before	253	0.4	3.6	61.7	34.4				3.0					
		After	735	1.2	7.3	79.6	11.8	<0.001	0.467	<0.001	3.7	<0.001		5.2		<0.001
16	4–15	Before	295	2.7	3.7	44.1	49.5				3.4					
		After	294	0.3	6.8	81.3	11.6	<0.001	0.038	<0.001	3.3	0.874		5.7		<0.001

1 χ^2^ test of proportions before vs after acquisition,

2 Fisher’s exact test,

3 Generalised linear model for samples/day assuming normal distribution.

## Analysis

### Interviews

Responses were extracted from open-ended questions and summarised, using content analysis (Table 4), and for questions in which alternative responses were presented (Table 2), we derived total numbers of respondents giving each answer.

**Table 4:** Recalled incidence of hypoglycaemic events before and after acquiring dog - in client’s own language and units.

Clientnumber	Estimated frequency of low blood sugar(i.e. below desired target range)		Estimated frequency unconscious		Estimated frequency of paramedic call outs	
	Pre-dog	Currently	Pre-dog	Currently	Pre- dog	Currently
1	several/day	several/day	6/year	0	0	0
2	several/week	0	2/month	0	*100s*	*1 (dog not present)*
3a	2/day	2/day	2	0	*1*	*0*
3b						
4	*2–3/week*	*1/month*	2–3/month	0	*8–9/year*	*1*
5	2–3/day	0s	*2 blackouts, 1 coma*	*2 (dog warned)*	1/wk when pregnant, then <10	0
6	*1/day*	*2*	3–4/week	0	*3 in 4 years*	*0*
7	*3–4/day*	*1/day*	2/day	0	*1/week*	*1/year*
8	3/day	3/day (not so low)	*never entirely - 2 collapses/week*	*0*	0	0
9	3–4/week	3–4/week	0	0	*4–5 in total*	*0*
10	*unknown*	*3–4/week*	*1/month*	*1*	*1/month*	*5 (dog not present during 3)*
11	*constant at worst, 3–4/day*	*1/day*	every 4–5 weeks, sometimes 2/night	0	2/night at worse	0 in 4.5 years
12	*5–6/day*	*4/week*	0	0	0	0
13	unsure as didn’t know until unconscious	10	*3–4/week*	*1/week*	*4/month*	*1*
14	didn’t test	4/day	2–3 in total a	1 (not sure diabetes)	2–3 in total	1
15	*all the time*	*2–3/day*	3–4/week	0	3–4/week	0
16	*4–5/week*	*3–4/week*	*8/year*	1*	*1/year*	*1* a

Underline represents incidents which never occurred post dog; italic represents incidents noted to decrease considerably in frequency post-dog.

a Unable to ascertain if reduced frequency due to short period of dog ownership.

### Comparing Alert and Routine Samples

Five clients failed to provide more than three days’ of post-dog routine and alert records, whilst 3a and 3b were combined as they used the same dog (Table 1). For the remaining clients, every available glucose concentration was classified as below, within or above the client’s individual target range. For each client in turn, we tested the null hypothesis that the distribution of values across these three categories was the same in routine and alert samples. If they were not, we concluded that the dog was not alerting randomly, but at a different rate when the clients glucose concentration was out of range. This conclusion depends on the assumption that glucose concentrations during routine and random samples would be similar; an assumption we were unable to test but which past research suggests is reasonable, since routine eight-point sampling has been shown to correlate well with continuous twenty-four hour glucose profiles [15]. The proportions of samples that were out of range were compared (between routine and alert samples) using logistic regression and the results presented as odds ratios with 95% confidence intervals.

### Glycamaeic Control

Pre-dog data could not be sourced for six clients whilst a further two clients failed to provide post-dog routine records, hence comparison of pre- and post-dog data was limited to nine clients (Table 1). We tested for change in the distributions of glucose concentration following dog acquisition (Chi-squared statistics for high/target/low x before/after placement), and specifically examined occurrence of very low (below 2.5 nm/l) and nocturnal lows (2200 hrs–0600 hrs). A global test for change following the placement of the dogs was conducted using Cochran-Mantel-Haenszel statistics.

We tested for change in HbA1c following dog acquisition, averaged over all pairings, and within individual pairings, using analysis of variance with normal error distributions.

## Results

### Interviews

When asked to recall the incidence of hypoglycaemia, currently and before having a trained dog, all clients reported a reduction in either frequency of low blood sugar, unconscious episodes or paramedic call outs and six clients believed all three had been reduced (Table 4). Eight people reported that they had never been unconscious since having a trained dog (although they had previously), whilst three reported paramedic call outs pre- but not post-dog acquisition.

When asked the extent to which they agreed with each of ten statements regarding the dog’s effect upon their life (Table 2), the majority of clients totally agreed that they were more independent post-dog (12/16), whilst two somewhat agreed and two clients were neutral. The other statements which prompted strong opinions were “The dog has enhanced my quality of life”, and “I am totally satisfied with my dog” with which 13 strongly agreed (2 somewhat agreed). The vast majority of respondents, strongly disagreed with the statement “If I had my time again, I wouldn’t get a dog” suggesting they would be keen to have a dog again, whilst opinions varied regarding whether the dog was a big commitment, and whether they liked or disliked the attention promoted by the dog’s charity coat. Almost all the clients (15), trusted their dog to alert them when their blood sugars were low and 13 also trusted them to alert when blood sugars were high (6 totally, 7 somewhat).

### Comparing Alert and Routine Samples

**Figure 1:**
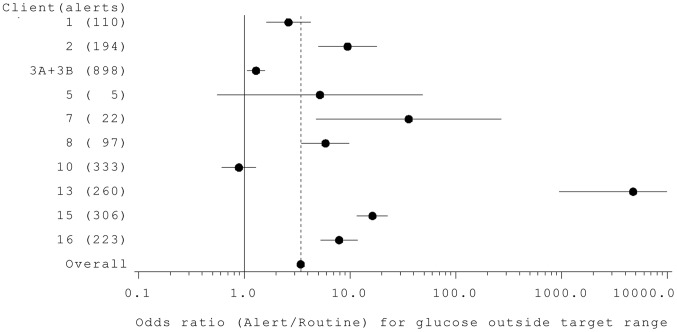
Odds ratios (Alert/Routine) for samples with glucose concentrations outside each client’s target range in alert samples and routine samples after the acquisition of their dog. Horizontal lines indicate 95% confidence intervals and any that do not span 1 so are significant at p<0.05; vertical dotted lines represents the overall estimate with all dogs pooled in a single analysis. The odds ratios for dog 3 were similar for its two clients, and the data have therefore been pooled.

Blood tests for 8 of 10 clients showed the odds of an alert sample being out of target range was significantly greater than that of a routine sample (p<0.05; Figure 1).The ratio for dog 5 was close to the average of 3.4, but with very wide confidence intervals reflecting the small amount of data available for this dog (only 5 alerts). Dog 10 is the only one apparently alerting at random, although there are possible explanations for this (see Discussion).

### Glycaemic Control

**Figure 2:**
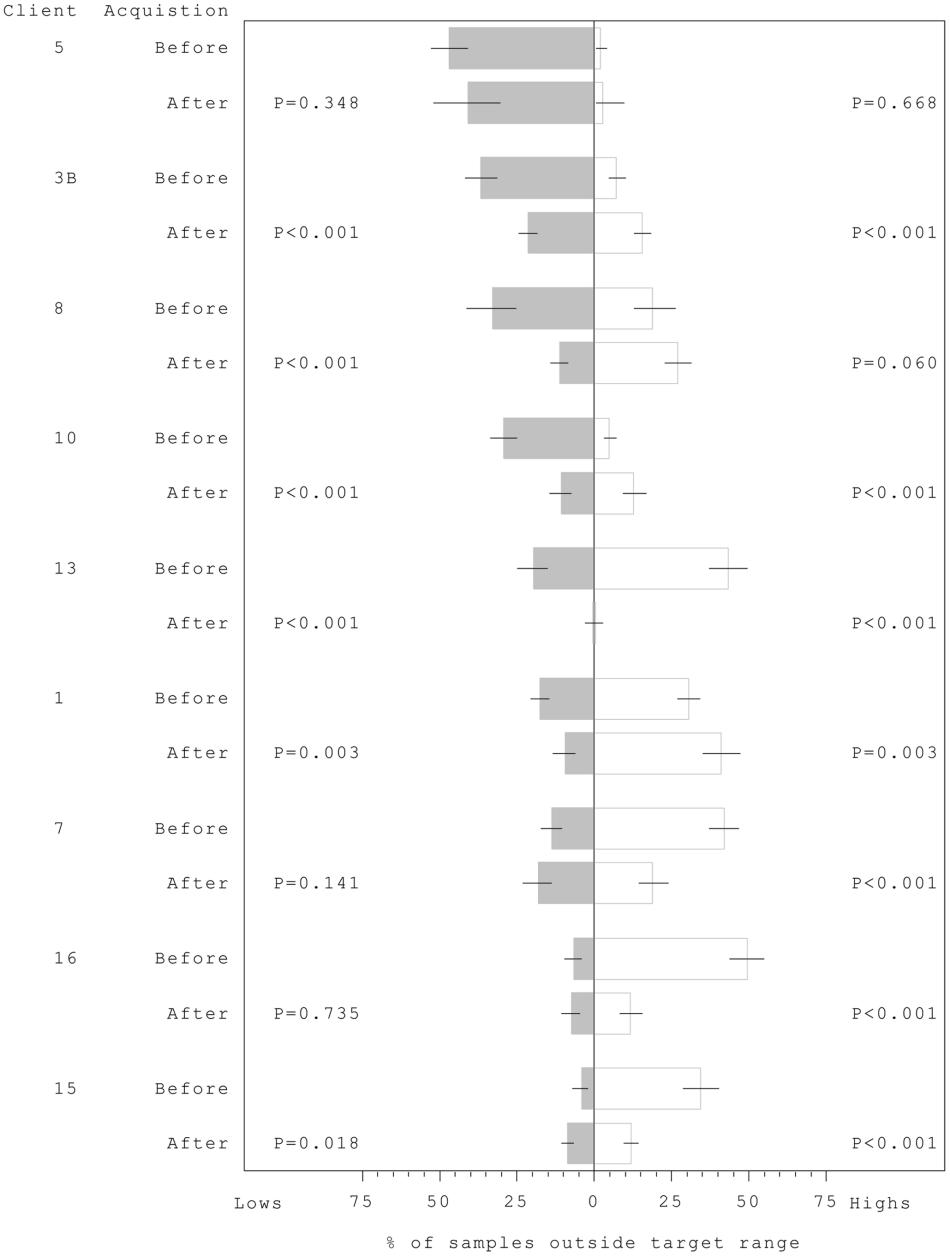
Percentage of routine samples with glucose concentrations above and below each client’s target, taken before and after acquisition of a trained dog. Clients presented in descending order of % low before acquisition and p values for test for significant change in proportion of readings below (left) and above target range (right).

Overall there was highly significant evidence of change occurring after dog acquisition (p<0.001). In eight out of nine cases, there was a significant shift in the distribution of glucose levels relative to the client’s target range following the placement of their dog (Table 3). The exception was pairing 5 which had limited post-dog data. In all cases, except pairing 1, there was an increase in the percentage of samples within target range post-dog, but the pattern of change differed between clients (Figure 2). In the six clients with the highest percentage of lows pre- dog (5, 3b, 8, 10, 12, 1), the percentage decreased after placement, significantly for all but pairing 5. In three cases this was accompanied by a significant increase in the percentage of highs. Pairing 13 is an exception with very few lows or highs after dog placement. The three remaining clients (7, 16, 15) had few pre-dog records below target, but more frequent above; post-dog records saw a significant decrease in frequency of these highs. Two clients recorded a significant decrease in “very low” values, post-dog. However, the overall frequency with which clients recorded blood tests differed with five clients recording significantly more frequently and three significantly less after dog acquisition (Table 3).

**Figure 3:**
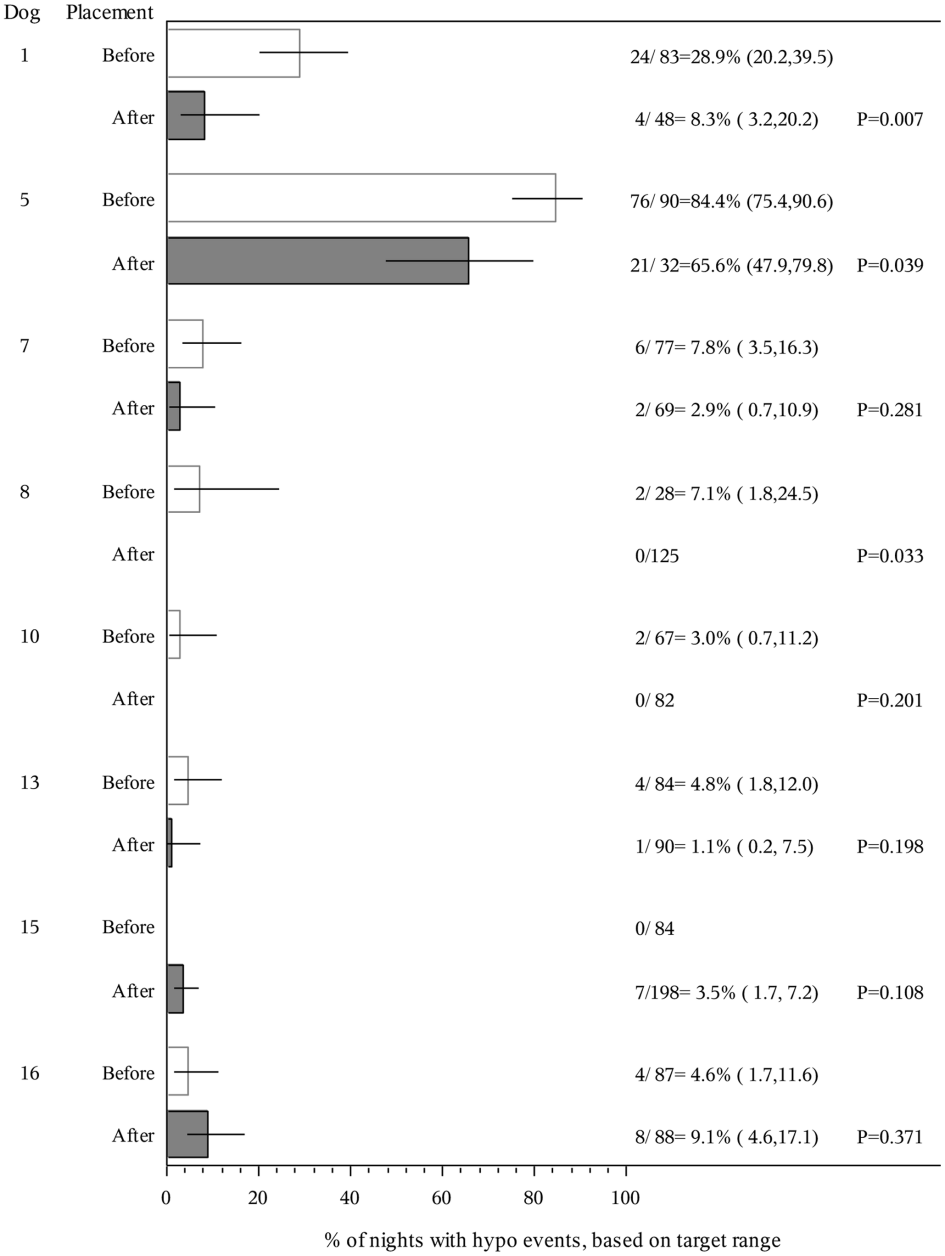
Percentage of nights in which blood glucose readings were recorded to be below clients’ target range before and after dog acquisition.

For the eight clients who recorded nocturnal lows pre-dog, and also provided post-dog data, we saw a reduction in the proportion of nights during which lows were recorded in six (two significantly; Figure 3), and an increase in two (one reaching statistical significance).

**Figure 4:**
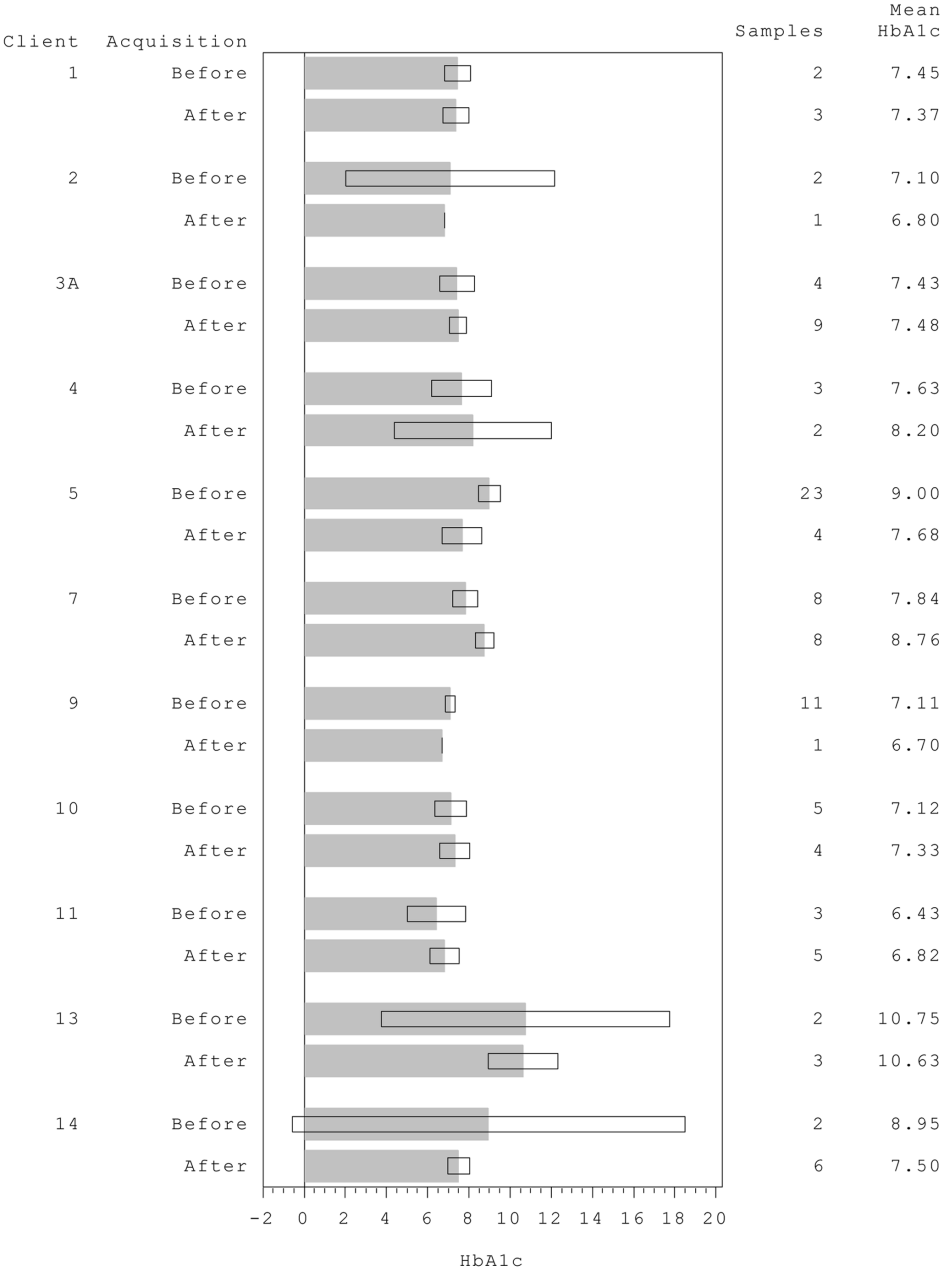
Mean % HbA1c (glycated haemoglobin), from six-monthly medical records before and after acquisition of trained alert dogs. Shaded bars represent means and white boxes, the 95% Confidence Intervals.

Hb1Ac overall showed a small and non-significant reduction following dog placement (least square means before and after: 7.89 (7.63, 8.15) and 7.75 (7.45, 8.04) respectively; Figure 4). There is evidence of heterogeneity between pairings (p = 0.024), with some clients showing relatively large reductions (e.g. clients 5 and 14), but there was too little data to test for a change in individual clients.

## Discussion

Acquisition of a trained alert dog was greatly valued by the majority of this self-selected sample of medical alert-dog users. They believed their dog to reliably alert to changes in blood sugar and hence described increased independence since obtaining the dog. The population, overall, reported reduced unconscious episodes and paramedic call outs, which if accurate, is of great importance since not only does it represent increased health and safety to the client, but also potentially significant reduced costs in health care.

Owner-recorded data support interview claims, since for 80% of the clients providing sufficient data, when their dog was recorded to perform an alerting behaviour their blood was significantly more likely to be out of target range than it was during routine samples. In addition, comparison of owner’s routine test records from before and after obtaining their dog, showed highly significant overall change: all but one client being more likely to be within target range post-dog; five out of nine clients experienced a significantly reduced incidence of low blood sugars, and three of the remaining four showed a significant reduction in high blood sugars, suggesting improved glycaemic control in most clients. The two clients who showed no significant increase in percentage within target (1 and 5), had dogs which were unqualified and the clients reported to be experiencing training problems, which were subsequently resolved.

Some clients experienced an increased frequency of either low or high blood sugars post-dog which may initially cause concern. However, this detailed data was collected over a too short time-frame, which in some cases coincided with a time of personal upheaval (e.g. client 5) or altered insulin regime (client 1). Blood test results also give us no information on the length of time for which each client remained above or below target level, just the incidence of such events, hence further data is required. For the three clients whose proportion of lows overall increased, the frequency of nocturnal lows decreased in one case (7) and the other two (15 and 16) were still trainee dogs. The time between the two data collection periods ranged from 5 to 581 months, dependent upon when the dog had been acquired. Although other changes may have occurred over the intervening period (e.g. changes in insulin regimes, doctors, stress, maturation) these are as likely to adversely affect gylcaemic control as they are to benefit control, hence they are unlikely to represent a consistent bias. For all those clients that recorded increased high-levels post-dog (Figure 2) and also provided HbA1c data (Figure 4), a decrease in HbA1c post-dog was noted, suggesting that over a longer time-frame, dog acquisition may have coincided with tightened glycaemic control.

In the population as a whole, there was very small reduction in HbA1c after obtaining a dog, although the number of records varied greatly between clients. Since most clients experienced a decrease in reported hypoglycaemic episodes, one may have expected an accompanying increase in time in hyperglycaemia, and hence this result is encouraging and further supports the idea of tightened control. However, the effect is small and varies with client. It seems anomalous, that despite a large reduction in the proportion of both highs and lows for client 13, their HbA1c appears little changed post-dog. Differing recording time-frames, and altered routine blood testing frequency after dog allocation (Table 3), may be responsible for this anomaly. Future work may benefit from using Continuous Glucose Monitoring to examine the duration within and outside target range, as well as using extended HbA1c records to explore longer-term glycaemic control.

Interviews showed that owners of trained dogs generally hold a very positive view of the dogs’ impact on their lives. When recalling hypoglycaemic events, every one of the fifteen interviewees (who had had the dog sufficiently long) reported a reduction in frequency of at least one of the parameters reported (paramedic call outs, hypoglycaemic, or unconscious episodes). Of course, memory recall may not be accurate, so future studies should be longitudinal, with subjects recording data at the time, and should source independent health care data whenever possible. However, the consistent trends emerging in clients’ reports and records in this study are unlikely to be an artefact.

Thirteen owners totally trusted their dog to alert them to low blood sugar, whereas only six expressed total trust in alerts to hyperglycaemia. This is unsurprising, given that the latter is a secondary task, trained subsequent to a strong alert to low blood sugar. Some of the less experienced and trainee dogs may not have been taught this at the time of interview. Opinions varied when clients rated the degree to which they enjoyed the attention and conversations promoted by the coat (which is bright red and states that the dog is a medical alert dog). This likely reflects differences in personality of the clients, with some being more extroverted and happy to engage with strangers. Differences in whether the dog was viewed as a big commitment are likely affected by whether the client had previously owned dog(s) or whether this is their first experience of dog ownership.

Psychosocial analysis of this population [14] suggests that strict selection criteria of the charity, and self-selection by clients, has resulted in an extreme population with highly brittle (unstable) diabetes. Clients show great fear of hypoglycaemia, [16], and a very high Average Weighted Impact of diabetes on their Quality of life [17]. Despite this, their present Quality of Life and Wellbeing [18] are comparable to other populations of non-dog users living with Type I diabetes [19]. This suggests that the benefits of alert-dog ownership reported here have improved the clients’ life quality to levels comparable to the general Type I diabetes population.

### Is there Evidence that Alert Samples were more Likely to be out of Range than Routine Samples, and does this Vary between Dogs?

Most dogs showed a significantly higher proportion of alerts when their owners’ blood sugars were out of target range than within target range, indicating that alerting behaviour is unlikely to be random. In the case of the best performing dog, the odds of an alert being when bloods were out of range were 10,000 times higher than that of routine tests.

There is apparent marked variation in dog performance. We suggest a number of potential reasons for this:

- Individual dogs naturally vary in their aptitude for the task; whilst some dogs were specifically chosen for their potential to work as a glycaemia alert dog, others were clients’ pets which have been trained in situ with support from Medical Detection Dogs. Aptitude varies between individuals and between breeds in many other detection dog roles [20] so optimal dog selection is critical.
- Some of the apparent shortfalls in performance may be accounted for by deficiencies in the blood glucose data collection methodology. Eleven clients (including those with the apparently least reliable dogs (10 and 5)) described their dog to often alert when their blood sugars were within range although subsequent blood tests reveal they had in fact been rapidly dropping. This phenomenon warrants additional research; studies including Continuous Glucose Monitoring System with continual traces will, in the future, help elucidate the efficiency with which dogs can detect falling sugars, ahead of them reaching below target levels.
- Record-keeping by owners is unlikely to be perfect: some alerts may have been missed, and some alerts incorrectly recorded as routine tests. Especially in the case of dog 3, which is trained to alert two clients, the dog may not have always been present at times of 3b’s altered blood sugar and the clients may not have accurately reported this.
- Since the data is owner-reported, it is also possible that a belief in their dogs’ ability led some owners, consciously or inadvertently, to be more likely to record ambiguous behaviours as alerts only when their sugars were subsequently found to be low, as compared to normal, or to conduct routine tests, when they were likely to be in within target range. However clients were made aware that records were also to be used to detect training issues and to direct remedial training, so we believe this risk was mitigated and it is extremely unlikely that all clients were biased. Future studies with owner-independent measures of ability are therefore critical to fully ascertain dogs’ accuracy. Further studies involving remote recording show considerable promise; a very small pilot trial, used CGMS and objective third party observations of dog behaviour to eliminate self-reporting and subjective interpretation (Rooney et al unpublished data). This appears to support the current findings that dogs’ behaviour is affected by their owners’ glucose levels.
- Initial training is done by, or under the supervision of, the charity, but dogs live in close association with their owners and are heavily influenced by their behaviour, including their ability to effectively reward correct alerts. Owners vary in their training ability, due to differences in their willingness and ability to follow instructions, their past experience with dogs, their mood, and their behaviour when their blood sugars fall. For example some people become un-responsive, which can be aversive to the dog. Training method choice has been shown to affect dog performance (e.g. [21], [22], [23]), and although clients are trained to use reward-based training, subtle differences and inconsistencies in style may in turn affect their dog’s ability to carry out its trained task.

### How do Dogs Respond to Change in Blood Sugar Level?

Dogs have been shown to respond when their owners’ sugars are low or high, but as yet we cannot be sure as to what they are actually responding. Odour cues are the most plausible explanation [24] especially as dogs show “alert” behaviours when their owners are asleep (e.g. [8]) and presumably emitting few behavioural cues (although changes in breathing rate may occur). In addition, owners frequently report their dogs responding when they are in another room and behavioural cues therefore implausible.

It is likely that dogs detect changes in the chemical composition of their owners’ sweat, or breath (including products of ketosis), using their acute sense of smell. This is supported by the fact that MDD are increasingly training new dogs using remote odour samples collected from clients during times of hypoglycaemia, before they introduce dog and owner. Unlike the training of seizure alert dogs [25], MDD do not intentionally train dogs to respond to behavioural cues. However, once placed some dogs may learn to utilise additional predictive cues as well as odour, including subtle changes in their owners’ mood or behaviour (e.g. trembling, becoming disorientated). Research is now required to determine the precise cues used and to identify any odour signature involved.

This study is the first to examine the effectiveness of trained glycaemia alert dogs and has demonstrated that most clients are willing and able to collect data, although some improvement in recording methods is recommended. Although based mainly on owner-recorded data, multiple findings point consistently to the potential value of trained alert dogs, but for conclusive proof, longitudinal studies are now required, examining matched clients pre- and post-dog allocation. Such studies can never be truly randomised, as the population willing to use a dog as an intervention will by necessity be self-selected. However, comparison of waiting list applicants to those who have acquired a trained dog, will help to determine the full value of this intervention.
